# A Real-World Comparative Analysis of Lenvatinib and Sorafenib as a Salvage Therapy for Transarterial Treatments in Unresectable HCC

**DOI:** 10.3390/jcm9124121

**Published:** 2020-12-21

**Authors:** Jaejun Lee, Pil Soo Sung, Hyun Yang, Soon Kyu Lee, Hee Chul Nam, Sun Hong Yoo, Hae Lim Lee, Hee Yeon Kim, Sung Won Lee, Jung Hyun Kwon, Jeong Won Jang, Chang Wook Kim, Soon Woo Nam, Si Hyun Bae, Jong Young Choi, Seung Kew Yoon

**Affiliations:** 1 The Catholic University Liver Research Center, College of Medicine, The Catholic University of Korea, Seoul 06591, Korea; pwln0516@gmail.com (J.L.); oneggu@naver.com (H.Y.); blackiqq@gmail.com (S.K.L.); hcnam128@catholic.ac.kr (H.C.N.); calla0108@naver.com (S.H.Y.); atom069@naver.com (H.L.L.); hee82@catholic.ac.kr (H.Y.K.); zambrotta@catholic.ac.kr (S.W.L.); doctorkwon@catholic.ac.kr (J.H.K.); garden@catholic.ac.kr (J.W.J.); cwkim@catholic.ac.kr (C.W.K.); drswnam@hanmail.net (S.W.N.); baesh@catholic.ac.kr (S.H.B.); jychoi@catholic.ac.kr (J.Y.C.); yoonsk@catholic.ac.kr (S.K.Y.); 2 Division of Gastroenterology and Hepatology, Department of Internal Medicine, College of Medicine, Eunpyeong St. Mary’s Hospital, The Catholic University of Korea, Seoul 03382, Korea; 3 Division of Gastroenterology and Hepatology, Department of Internal Medicine, College of Medicine, Seoul St. Mary’s Hospital, The Catholic University of Korea, Seoul 06591, Korea; 4 Division of Gastroenterology and Hepatology, Department of Internal Medicine, College of Medicine, Incheon St. Mary’s Hospital, The Catholic University of Korea, Seoul 22711, Korea; 5 Division of Gastroenterology and Hepatology, Department of Internal Medicine, College of Medicine, Bucheon St. Mary’s Hospital, The Catholic University of Korea, Seoul 14647, Korea; 6 Division of Gastroenterology and Hepatology, Department of Internal Medicine, College of Medicine, Uijeongbu St. Mary’s Hospital, The Catholic University of Korea, Seoul 11765, Korea

**Keywords:** hepatocellular carcinoma, lenvatinib, sorafenib, objective response

## Abstract

Background/Aims: Lenvatinib was recently approved as a first-line oral multikinase inhibitor for unresectable hepatocellular carcinoma (HCC). In this study, we aimed to compare the efficacy and safety of lenvatinib and sorafenib for the treatment of unresectable HCC in patients with prior failure of transarterial treatment. Methods: Between January 2019 and September 2020, 98 unresectable HCC patients treated with lenvatinib or sorafenib as salvage therapy were enrolled from five Korean university-affiliated hospitals. Progression-free survival (PFS), overall survival (OS), objective response rate (ORR), and disease control rate were calculated to assess the antitumor response. Results: A total of 43 and 55 patients were treated with lenvatinib and sorafenib, respectively, as salvage therapy after the failure of transarterial treatments. The median PFS was 4.97 months in the lenvatinib group and 2.47 months in the sorafenib group (*p* = 0.001, log-rank test). The ORR was significantly higher in the lenvatinib group (25.6%) than in the sorafenib group (3.6%, *p* = 0.002). Use of lenvatinib over sorafenib (hazard ratio: 0.359, 95% confidence interval: 0.203–0.635, *p* < 0.001) was the most significant factor for a favorable PFS after the failure of transarterial treatments in all enrolled patients. For favorable OS, achieving objective response was the significant factor (hazard ratio 0.356, 95% confidence interval: 0.132–0.957, *p* = 0.041). There were no significant differences in the safety profile between the two groups. Conclusions: In this real-world study, lenvatinib was demonstrated to be more efficacious than sorafenib as a salvage therapy for transarterial treatments in unresectable HCC.

## 1. Introduction

Liver cancer is the second leading cause of cancer death worldwide and mainly comprises hepatocellular carcinoma (HCC) cases [1,2]. The standard treatment for intermediate to advanced HCC has been controversial due to varying tumor burdens and different liver reserves [3]. Traditionally, Barcelona Clinic Liver Cancer (BCLC) stage B or C HCC cases that are not suitable for surgical resection have been treated with transarterial methods such as transarterial chemoembolization (TACE), transarterial radioembolization (TARE), or hepatic arterial infusion chemotherapy (HAIC) [4,5,6]. Patients with TACE-refractory HCC usually have been considered suitable for multikinase inhibitor treatment, and sorafenib has been used as the standard salvage treatment since the SHARP trial in 2008 [6,7]. Recently, several multikinase inhibitors have been developed, but they have failed to show superiority or non-inferiority to sorafenib and/or failed to show superiority to placebo controls [8,9,10].

Lenvatinib is a multikinase inhibitor that has recently been demonstrated to be non-inferior to sorafenib in a phase 3 randomized controlled trial (the REFLECT trial) [11]. Since its approval as a first-line oral multikinase inhibitor, several real-world studies on the efficacy and safety of lenvatinib as a first-line treatment for advanced HCC have been performed worldwide [12,13,14]. Due to the heterogeneity of intermediate to advanced HCC, some studies have proposed the use of subgroup analyses for lenvatinib efficacy [15,16,17,18]. A recent report demonstrated that lenvatinib may be useful as an initial treatment for multinodular intermediate stage HCC exceeding the up-to-seven criteria in patients with good liver function who are expected to fail to attain a good prognosis after transarterial treatments [13,19,20]. However, in Korea, a large percentage of patients are treated with molecular targeted agents as salvage therapies after loco-regional treatments such as TACE. In addition, there are limited data that provide a head-to-head comparison between lenvatinib and sorafenib as salvage treatments after the use of other treatment modalities. Due to the insurance coverage issues, the efficacy and safety of lenvatinib have not been studied widely in Korea until recently. This retrospective, multicenter study was designed to elucidate the clinical efficacy and safety of lenvatinib as a salvage therapy after transarterial treatments and to compare them with those of sorafenib in an area with endemic hepatitis B virus (HBV) infection.

## 2. Patients and Methods

### 2.1. Patients

This was a retrospective, multicenter study conducted at five Korean centers. The Institutional Review Board of the Catholic University of Korea approved this study (XC20RIDI0059). This study was performed in accordance with the ethical standards of the institutional research committee and the most recent revision of the Declaration of Helsinki. The need for informed consent in this study was waived due to the retrospective nature of the analyses. Between January 2019 and September 2020, all patients diagnosed with histologically or clinically confirmed unresectable HCC (uHCC) according to the latest international guidelines who received lenvatinib or sorafenib treatment after the failure of transarterial treatments were retrospectively analyzed [3,21,22]. In the present study, we defined cases as “TACE-refractory cases” if any of the following conditions were met: (1) Insufficient necrotic area or an increase in viable tumor size after ≥2 consecutive TACE treatments; (2) increase in the tumor number or the development of new lesions during or within a few weeks of the TACE sessions; (3) persistent elevated levels of tumor markers after TACE; and (4) new development of vascular invasion or extrahepatic spread during or within a few weeks of the TACE sessions [23,24]. Since there is no precise definition for “HAIC refractory”, we defined it to be ‘showing more than 20% increase in the maximal diameter of the tumor (whether the tumor is hypervascular or infiltrative) or appearance of new intrahepatic or extrahepatic lesions after HAIC [25]. We performed response evaluation in every two cycles of HAIC.

**Figure 1:**
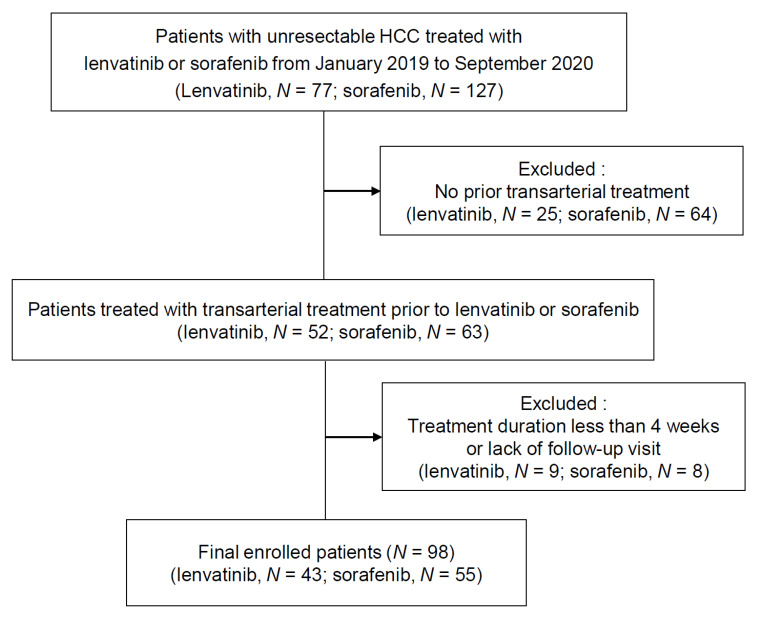
Patient population.

The inclusion criteria for the present study were as follows: (1) Confirmed intermediate to advanced HCC showing an insufficient response to transarterial treatments (TACE, TARE, or HAIC); (2) age ≥18 years; and (3) Eastern Cooperative Oncology Group (ECOG) performance status 0 or 1. The exclusion criteria were as follows: (1) Lack of follow-up visits after the start of the treatment; (2) a treatment duration of less than 4 weeks; (3) a Child-Pugh score >7; and (4) a history of malignancy other than HCC in the previous 5 years. A flow diagram of patient enrollment is shown in Figure 1. Initially, 77 patients who received lenvatinib treatment and 127 who received sorafenib treatment were reviewed. After the exclusion of patients who had not received prior transarterial treatment, 52 and 63 patients who received lenvatinib and sorafenib treatment, respectively, were included. Finally, we excluded patients without follow-up visits or with a treatment duration of less than 4 weeks. Clinical findings after the introduction of lenvatinib and sorafenib were retrospectively reviewed by experienced hepatologists. 

### 2.2. Lenvatinib and Sorafenib Treatment

In our centers, patients with sufficient hepatic reserve (Child-Pugh score ≤ 7) and BCLC stage B or C disease are usually selected to receive sorafenib or lenvatinib. Lenvatinib was administered once daily at a dose of 8 mg for patients weighing <60 kg and at a dose of 12 mg for patients weighing ≥60 kg. The sorafenib dose was decided by the clinician considering the patient’s age, ECOG performance status, body weight, and liver reserve; most patients were started on a dose of 400 mg, administered twice daily.

### 2.3. Evaluation of Treatment Responses and Assessment of Adverse Events

Radiologic responses were determined by two certified radiologists and classified according to the modified Response Evaluation Criteria in Solid Tumors (mRECIST) [26,27,28]. The first response evaluation was performed using computed tomography or magnetic resonance imaging (MRI) 4–8 weeks after drug administration, and further evaluations were performed every 8 weeks thereafter. Complete response (CR) and partial response (PR) were defined as the “disappearance of any intratumoral arterial enhancement in all target lesions” and as “at least a 30% decrease in the sum of the diameters of the viable target lesions,” respectively [26]. Adverse events (AEs) were assessed according to the Common Terminology Criteria for Adverse Events, version 4.0 [29]. Hand-foot skin reactions, proteinuria, hypertension, decreased appetite, and gastrointestinal symptoms such as diarrhea were identified by reviewing patient medical records.

### 2.4. Statistical Analysis

The median clinical parameter values were calculated, and their respective ranges were documented. Comparisons between groups were performed using a Student’s t-test when appropriate. Progression-free survival (PFS) was calculated from the start of the treatment to the date of disease progression, and drug cessation due to any cause in the absence of disease progression was censored. Overall survival (OS) was calculated from the start of treatment to the date of death or last follow-up and was estimated using the Kaplan–Meier method. Risk stratification was performed using the log-rank test. Cox regression analysis was performed to identify the factors that can affect survival outcomes. The objective response rate (ORR) was defined as the proportion of patients who achieved a CR and PR. The disease control rate (DCR) was defined as the proportion of patients with CR, PR, and stable disease. The therapeutic efficacies of lenvatinib, as demonstrated by the ORR and DCR, in patients with different characteristics were compared using Fisher’s exact test.

## 3. Results

### 3.1. Baseline Characteristics

We enrolled 43 uHCC patients who received lenvatinib (median age, 60 years; male:female, 35:8) and 55 uHCC patients who received sorafenib (median age, 63 years; male:female, 42:13); all patients had shown failure of transarterial treatments. There was no significant difference in the cause of HCC between the lenvatinib and sorafenib groups (Table 1). In the lenvatinib group, 8 and 35 patients were classified to have BCLC stage B and C disease, respectively, at the time of drug initiation. In the sorafenib group, 8 and 47 patients were classified to have BCLC stage B and C disease, respectively. All patients in the lenvatinib group were classified to have Child-Pugh class A (37 patients) or B (score ≥ 7; 8 patients) disease. There were no significant differences in Child-Pugh scores between the lenvatinib and sorafenib groups (*p* = 0.289). Previous treatments included the following: Lenvatinib group = TACE, 40 patients; HAIC, 8 patients and sorafenib group = TACE, 54 patients and HAIC, 4 patients. There was no difference in the frequency of radiological vascular invasion or the level of alpha-fetoprotein (AFP) between the groups; however, the sorafenib group had a lower median value of protein induced by vitamin K absence-II (PIVKA-II) than that of lenvatinib group (*p* = 0.020) (Table 1). Twenty-one of 43 patients treated with lenvatinib received a daily dose of 12 mg, and 22 patients received a daily dose of 8 mg. In the sorafenib group, only one patient (1.8%) was started with a dose of 400 mg a day, while others (98.2%) were started with 800 mg daily dosage of sorafenib.

### 3.2. Treatment Responses

At the time of the best response evaluation based on the mRECIST, 1 patient exhibited a CR and 10 exhibited a PR after lenvatinib introduction; only 2 patients showed a PR after sorafenib administration (Table 2). The ORR in the lenvatinib group was higher than that in the sorafenib group (25.6% vs. 3.6%, *p* = 0.002). Furthermore, the DCR was significantly higher in the lenvatinib group than in the sorafenib group (58.1% vs. 23.6%, *p* = 0.001).

### 3.3. Survival Outcomes

**Figure 2:**
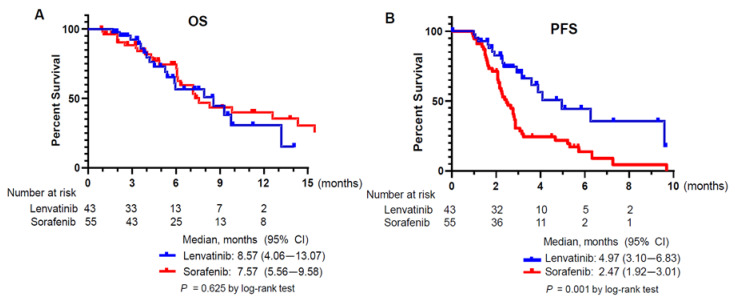
Kaplan Meyer survival curves of the patients. (**A**) Overall survival (OS) of the lenvatinib and the sorafenib group. (**B**) Progression-free survival (PFS) of the lenvatinib and the sorafenib group.

At the time of survival assessment, 18 of 43 (41.9%) patients in the lenvatinib group and 29 of 55 (52.7%) patients in the sorafenib group died. The median follow-up duration (lenvatinib: 4.50 vs. sorafenib: 5.73 months, *p* = 0.100), treatment duration (lenvatinib: 2.7 vs. sorafenib: 2.27 months, *p* = 0.174), and time to the first response evaluation (lenvatinib 1.80 vs sorafenib 2.03 months, *p* = 0.139) did not differ between two groups. The median OS from the start of lenvatinib or sorafenib was 8.57 and 7.57 months, respectively; this difference was not statistically significant (*p* = 0.625) (Figure 2A). Median PFS was longer in the lenvatinib group than in the sorafenib group according to a log-rank test (4.97 vs. 2.47 months; *p* = 0.001) (Figure 2B).

### 3.4. Adverse Events

During the treatment, there were 17 cases of Grade 3 AEs in the lenvatinib group, and there were no significant differences in the prevalence of severe AEs (grade 3 or higher) between the lenvatinib and sorafenib groups (17 vs. 18 cases, *p* = 0.592) (Table 3). In the lenvatinib group, hand-foot skin reaction was the most common event (13 documented cases), followed by proteinuria (10 documented cases). Other confirmed AEs included decreased appetite (*n* = 7), diarrhea (*n* = 5), hypertension (*n* = 4), fatigue (*n* = 3), increased bilirubin levels (*n* = 2), and hepatic encephalopathy (*n* = 2). For severe AEs (Table 3), hand-foot skin reaction (*n* = 4) and hypertension (*n* = 4) were most common in the lenvatinib group. In the sorafenib group, the most frequently observed adverse events were HFSR (*n* = 16), diarrhea (*n* = 11) and decreased appetite (*n* = 8). HFSR was found to be most common severe AEs (*n* = 8) in the sorafenib group.

### 3.5. Factors Contributing to Survival Outcomes

The factors associated with PFS in all enrolled patients (treated with lenvatinib + sorafenib) are shown in Table 4. In univariate analysis, use of lenvatinib over sorafenib, albumin-bilirubin (ALBI) grade 1, absence of macrovascular invasion, and AFP level less than 1000 ng/mL were shown to be good prognostic factors for a favorable PFS. However, use of lenvatinib over sorafenib (hazard ratio: 0.359, 95% confidence interval: 0.203–0.635, *p* < 0.001) was the only factor significantly associated with a favorable PFS in multivariate analyses (Table 4). We performed subgroup analyses for patients with lenvatinib treatment. A maximum tumor size of less than 5 cm was identified as an independent factor associated with a longer OS by log-rank test in patients with lenvatinib treatment (Figure S1).

Next, multivariate analyses were performed to find out prognostic factors for favorable OS in all enrolled patients (Table 5). The analyses were conducted in two models using different parameters to avoid interactions between independent variables. In multivariate model 1, achieving objective response was the significant prognostic factor contributing to prolonged OS (hazard ratio 0.356, 95% confidence interval: 0.132–0.957, *p* = 0.041). ALBI grade 1 was also shown to be associated with favorable OS. In multivariate model 2 that included the treatment choice variable (lenvatinib vs. sorafenib), ALBI grade 1 was the only factor shown to be associated with the prolonged OS.

### 3.6. A representative Patient Case

A 65-year-old male patient with chronic HBV infection was diagnosed with multinodular HCC. He was treated with TACE eight times from October 2018 to February 2020. Despite multiple rounds of TACE, new intrahepatic lesions developed persistently without extrahepatic metastasis or vascular invasion (Figure S2A). As the patient showed preserved liver function, based on a Child-Pugh score of 5 and an ALBI grade of 1, lenvatinib at a dose of 12 mg was initiated as salvage therapy. He experienced grade 3 hypertension, and anti-hypertensive drugs were administered. At the first evaluation after a treatment duration of approximately 2 months, intrahepatic lesions became nearly undetectable (Figure S2B).

## 4. Discussion

To our knowledge, this is the first study to involve a head-to-head comparative analysis on lenvatinib and sorafenib as a salvage treatment after transarterial treatments for advanced HCC in Korea. Lenvatinib appears to be safe and efficacious and shows an ORR superior to that of sorafenib in this setting. Lenvatinib administration resulted in a longer PFS (4.97 months) than sorafenib administration (2.47 months). Moreover, the use of lenvatinib over sorafenib was the most significant factor associated with favorable PFS after the failure of transarterial treatments in all enrolled patients. In this study, lenvatinib showed a significantly higher ORR and DCR (25.6% and 58.1%, respectively) than sorafenib (3.6% and 23.6%, respectively), although there was no significant difference in OS between the lenvatinib and sorafenib groups due to the small sample size and limited observational period. We expect that a study with a longer follow-up duration and a larger sample size may demonstrate the survival advantage of lenvatinib treatment over sorafenib treatment in salvage settings.

Lencioni et al. [30] emphasized that the objective response may be a potential predictor of OS in advanced HCC. Another study analyzed survival and objective response in a phase III study of lenvatinib (the REFLECT trial) and reached a similar conclusion [31]. In line with these studies, our study showed that achieving objective response was a significant factor for prolonged OS in multivariate Cox regression analysis for patients treated with lenvatinib or sorafenib, supporting the correlation between tumor responses and the prolonged OS. Moreover, in this study, preserved liver function (ALBI grade 1) was also associated with favorable OS, which was also mentioned in previous studies [15,32]. In our study, there were no statistically significant differences in the ORR and DCR between patients treated with lenvatinib after transarterial treatment failure and those treated without prior transarterial treatment (*n* = 25, ORR 20%, DCR 48%).

TACE-refractory uHCC has become a worldwide issue. It has been suggested that in these patients, the antitumor effect of TACE is avoided through neo-angiogenesis, and both vascular endothelial growth factor (VEGF) and fibroblast growth factor (FGF) seem to be associated with the failure of TACE [13]. Lenvatinib is a novel anti-angiogenic, multikinase inhibitor that targets VEGF receptors 1–3, FGF receptors 1–4, platelet-derived growth factor receptors, c-KIT, and RET proto-oncogene products [33,34,35]. FGF signaling pathways are critical in cancer angiogenesis and are thought to underlie the mechanisms of escape from anti-VEGF agents, and sorafenib targets it very weakly [36]. The differences in the types and intensities of targeting the kinases between sorafenib and lenvatinib might widen the gap between treatment response in HCC patients undergoing transarterial treatment.

In this study, the survival outcomes with sorafenib treatment were similar to those reported by other real-world studies in Korea and Japan [27,37]. However, there were large differences in survival outcomes with lenvatinib treatment between our real-world data and those reported in the REFLECT trial (PFS 7.4 months, ORR 24.1%, DCR 75.5%). This difference can be explained by the differences in the baseline characteristics of the participants [11]. The REFLECT trial included treatment-naïve patients and had a greater proportion of patients with BCLC stage B disease (22%) than our study. We also included many patients who were not eligible for the REFLECT trial, including those with major portal vein invasion, bile duct invasion, Child-Pugh class B disease, and a tumor occupying >50% of the liver volume. Therefore, our data reflect the “real-world” efficacy of lenvatinib more accurately. Recently, another real-world study on lenvatinib in Korea was published [14]. In that study, the median PFS and OS were 4.1 and 6.4 months, respectively [14], which are similar to the findings of this study. Shimose et al. [37] also reported a retrospective study comparing treatment efficacy of lenvatinib and sorafenib in TACE-refractory patients. The survival outcomes in that study were comparable to those in our study; PFS 5.8 vs 3.2 months, in lenvatinib and sorafenib group, respectively [37].

As presented in this study, lenvatinib can be safely and effectively administered. While lenvatinib showed better efficacy than sorafenib in terms of tumor response, the present study showed no difference in safety profiles between the two drugs. Most of the AEs in the lenvatinib group were manageable. Safety issues of lenvainib will be getting more important because multiple trials are now testing a combination of multikinase inhibitors and immune checkpoint inhibitors [38,39,40]. As the AEs associated with lenvatinib treatment are manageable, lenvatinib is a potential candidate for use in various combination treatments. Recently, a phase Ib study of lenvatinib plus pembrolizumab in patients with uHCC showed promising efficacy and manageable AEs [38].

In Korea, there is a government-covering reimbursement issue that the second-line treatment for sorafenib (regorafenib) is usually reimbursed and that for lenvatinib (in many cases, sorafenib) is not reimbursed, yet. Therefore, choosing lenvatinib or sorafenib is somewhat determined by the financial status of the patient because the cost for the second line treatment after lenvatinib is paid by the patient. Other issues are the etiology of HCC and tumor biology. Gardini et al. [41] stated that there is a clear trend that HCC patients with HBV infection have survival benefit in lenvatinib treatment over sorafenib treatment. There are also some reports describing that lenvatinib treatment may result in the objective response in some cases of aggressive HCC because it has unique immune modulatory effects and FGFR signaling blocking activity. In cases of highly proliferative HCC, usually, sorafenib seems to be have a very weak efficacy, although lenvatinib may result in the dramatic early responses [27,42].

This study has several limitations. First, this was a retrospective study. Second, lenvatinib has only recently been approved in Korea. Therefore, the sample size was small, and the observation period was short. In addition, further subclassification is needed because the present study included patients with both advanced and intermediate stage HCC at diagnosis. The use of propensity score matching would help clarify the difference in efficacy between sorafenib and lenvatinib. Further prospective studies with larger populations and longer observational periods on lenvatinib efficacy in patients who failed transarterial treatments are needed to demonstrate the efficacy of lenvatinib as a salvage treatment.

## Figures and Tables

**Table 1:** Baseline characteristics of the enrolled patients who received lenvatinib or sorafenib as a salvage treatment after transarterial treatment failure.

Parameters	Lenvatinib (*N* = 43)	Sorafenib (*N* = 55)	Total (*N* = 98)	*p*
Epidemiology				
Sex, M/F (%)	35 (81.4)/8 (18.6)	42 (76.4)/13 (23.6)	77 (78.6)/21 (21.4)	0.723
Age, median (range)	60 (32–85)	63 (43–86)	62.5 (32–86)	0.451
Etiology				0.881
HBV, *n* (%)	31 (72.1)	42 (76.4)	73 (74.5)	
HCV, *n* (%)	3 (7.0)	2 (3.6)	5 (5.1)	
Alcohol, *n* (%)	7 (16.3)	8 (14.5)	15 (15.3)	
Others, *n* (%)	2 (4.7)	3 (5.5)	5 (5.1)	
Child-Pugh score				0.289
5, *n* (%)	24 (55.8)	37 (67.3)	61 (62.2)	
6, *n* (%)	13 (30.2)	15 (27.3)	28 (28.6)	
7, *n* (%)	6 (14.0)	3 (5.5)	9 (9.2)	
BCLC stage				0.792
A, *n* (%)	0 (0)	0 (0)	0 (0)	
B, *n* (%)	8 (18.6)	8 (14.5)	16 (16.3)	
C, *n* (%)	35 (81.4)	47 (85.5)	82 (83.7)	
D, *n* (%)	0 (0)	0 (0)	0 (0)	
ECOG				0.942
0, *n* (%)	16 (37.2)	22 (40.0)	38 (38.8)	
1, *n* (%)	27 (62.8)	33 (60.0)	60 (61.2)	
AFP, median (range), ng/mL	278.9 (1.4–115807)	708.8 (1.3–512682)	647.4 (1.3–512682)	0.463
PIVKA-II, median(range), mAU/mL	806 (11–300000)	532.3 (14–52576)	638.3 (11–300000)	0.020
Macrovascular invasion, *n* (%)	19 (44.2)	25 (45.5)	44 (44.9)	1.000
Extrahepatic metastasis, *n* (%)	24 (55.8)	39 (70.9)	63 (64.3)	0.182
Previous treatments				
TACE, *n* (%)	40 (93.0)	54 (98.2)	94 (95.9)	
HAIC, *n* (%)	8 (20.9)	4 (7.3)	12 (12.2)	
Radiation therapy, *n* (%)	13 (30.2)	14 (25.5)	27 (27.6)	
Surgical resection, *n* (%)	5 (11.6)	9 (16.4)	14 (14.3)	
Radiofrequency ablation, *n* (%)	5 (11.6)	8 (14.5)	13 (13.3)	
Systemic chemotherapy, *n* (%)	5 (11.6)	1 (1.8)	6 (6.1)	

HBV, Hepatitis B virus; HCV, Hepatitis C virus; AFP, Alpha-fetoprotein; PIVKA-II, Protein induced by vitamin K absence-II; TACE, Transarterial chemoembolization; HAIC, Hepatic arterial infusion chemotherapy.

**Table 2:** Treatment response in the enrolled patients.

Treatment Response	Lenvatinib, *n* (%)	Sorafenib, *n* (%)	*p*
CR	1 (2.3)	0 (0)	
PR	10 (23.2)	2 (3.6)	
SD	14 (32.6)	11 (20.0)	
PD	14 (32.6)	35 (63.6)	
NA (Not Assessed)	4 (9.3)	7 (12.7)	
ORR	25.6	3.6	0.002
DCR	58.1	23.6	0.001

uHCC, unresectable hepatocellular carcinoma; CR, complete response; PR, partial response; SD, stable disease; PD, progressive disease; ORR, objective response rate; DCR, disease control rate.

**Table 3:** Grade ≥ 3 AEs associated with lenvatinib or sorafenib treatment.

Adverse Event	Lenvatinib, *n* (%)	Sorafenib, *n* (%)	*p*
AE grade ≥3	17	18	0.529
HFSR	4 (9.3)	8 (14.5)	
Proteinuria	3 (7.0)	0 (0)	
Hyperbilirubinemia	1 (2.3)	1 (1.8)	
Hepatic encephalopathy	2 (4.7)	2 (3.6)	
Diarrhea	2 (4.7)	2 (3.6)	
Hypertension	4 (9.3)	0 (0)	
Decreased appetite	1 (2.3)	3 (5.5)	
Elevated aspartate aminotransferase level	0 (0)	1 (1.8)	

AE, Adverse events; HFSR, Hand-foot-skin reaction.

**Table 4:** Univariate and multivariate analyses of the factors associated with favorable PFS in the total enrolled patients (lenvatinib + sorafenib).

	Univariate Analysis			Multivariate Analysis		
	HR	95% CI	*p*	HR	95% CI	*p*
**LEN vs SOR**	**0.399**	**0.230–0.692**	**0.001**	**0.359**	**0.203–0.635**	**<0.001**
Age (< 60 vs ≥ 60)	1.186	0.714–1.969	0.509			
ECOG (0 vs 1)	0.839	0.500–1.407	0.506			
**HBsAg positivity**	**1.573**	**0.850–2.911**	**0.149**	1.445	0.770–2.710	0.252
Tumor size (≤5cm)	0.928	0.557–1.547	0.928			
**ALBI grade 1**	**0.576**	**0.336–0.987**	**0.045**	**0.612**	**0.353–1.061**	**0.080**
**AFP (ng/mL) ≤1000**	**0.594**	**0.361–0.979**	**0.041**	0.719	0.406–1.272	0.257
**PIVKA-II (mAU/mL)** **≤100**	**0.592**	**0.319–1.098**	**0.097**	0.702	0.346–1.426	0.328
Child Pugh score (5)	0.751	0.452–1.249	0.270			
**Macrovascular invasion**	**1.664**	**1.005–2.755**	**0.048**	1.302	0.729–2.327	0.372
Extrahepatic metastasis	0.932	0.577–1.655	0.977			

LEN, Lenvatinib; SOR, Sorafenib; ECOG, Eastern Cooperative Oncology Group; ALBI, Albumin-bilirubin grade; AFP, alpha-fetoprotein; HR, hazard ratio; CI, confidence interval; PIVKA-II, Protein induced by vitamin K absence-II. Significant variables are in bold characters.

**Table 5:** Univariate and multivariate analyses of the factors associated with favorable OS in the total enrolled patients (lenvatinib + sorafenib).

	Univariate Analysis		Multivariate Analysis			
			Model 1		Model 2	
	HR (95% CI)	*p*	HR (95% CI)	*p*	HR (95% CI)	*p*
**Objective Response**	**0.488 (0.192–1.238)**	**0.131**	**0.356 (0.132–0.957)**	**0.041**		
LEN vs SOR	1.166 (0.629–2.160)	0.625			0.771(0.390–1.524)	0.454
Age (<60 vs ≥60)	1.416 (0.782–2.552)	0.247				
ECOG (0 vs 1)	0.570 (0.305–1.065)	0.078	1.488 (0.678–3.264)	0.322	1.327 (0.599–2.938)	0.486
HBsAg positivity	0.776 (0.412–1.462)	0.432				
**Tumor size (≤5cm)**	**0.423 (0.232–0.771)**	**0.005**	0.557(0.292–1.065)	0.077	0.562 (0.289–1.091)	0.088
**ALBI grade 1**	**0.425 (0.215–0.838)**	**0.013**	**0.422 (0.186–0.959)**	**0.039**	**0.408 (0.179–0.930)**	**0.033**
**AFP (ng/mL)** ≤1000	**0.447 (0.248–0.808)**	**0.008**	0.668 (0.342–1.305)	0.238	0.629 (0.318–1.246)	0.184
**PIVKA-II (mAU/mL)** **≤100 **	**0.228 (0.088–0.592)**	**0.002**	0.336 (0.113–1.003)	0.051	0.336 (0.113–0.998)	0.050
**Child Pugh score (5)**	**0.553 (0.310–0.988)**	**0.045**	0.877 (0.412–1.867)	0.877	0.940 (0.432–2.046)	0.877
**Macrovascular invasion**	**2.365 (1.299–4.304)**	**0.005**	1.950 (0.960–3.963)	0.065	1.546 (0.788–3.034)	0.205
Extrahepatic metastasis	0.949 (0.517–1.742)	0.865				

LEN, Lenvatinib; SOR, Sorafenib; ECOG, Eastern Cooperative Oncology Group; ALBI, Albumin-bilirubin grade; AFP, alpha-fetoprotein; HR, hazard ratio; CI, confidence interval; PIVKA-II, Protein induced by vitamin K absence-II. Significant variables are in bold characters.

## Supplementary Materials

The following are available online at https://www.mdpi.com/2077-0383/9/12/4121/s1, Figure S1: Overall survival of the patients in the lenvatinib group according to the tumor size. Figure S2: A representative patient case who obtained partial response with lenvatinib after failure of multiple rounds of TACE.

## Abbreviations

HCC: Hepatocellular carcinoma; BCLC: Barcelona-Clinic Liver Cancer stage; TACE: transarterial chemoembolization; TARE: transarterial radioembolization; HAIC: hepatic arterial infusion chemotherapy; HBV: Hepatitis B virus; uHCC: unresectable hepatocellular carcinoma; ECOG: Eastern Cooperative Oncology Group; mRECIST: modified Response Evaluation Criteria in Solid Tumors; MRI: magnetic resonance imaging; CR: Complete response; PR: partial response; SD: Stable disease; PD: Progressive disease; AE: Adverse event; PFS: Progression-free survival; OS: Overall survival; ORR: Objective response rate; DCR: Disease control rate; AFP: alpha-fetoprotein; PIVKA-II: protein induced by vitamin K absence-II; ALBI: Albumin-bilirubin; CHB: Chronic hepatitis B; VEGF: vascular endothelial growth factor; FGF: fibroblast growth factor; PDGF: platelet-derived growth factor
