# Supplementary material for: A Real-World Comparative Analysis of Lenvatinib and Sorafenib as a Salvage Therapy for Transarterial Treatments in Unresectable HCC
Source: J Clin Med. 2020 Dec 21;9(12):4121. doi: 10.3390/jcm9124121 (PMC7767204; doi:10.3390/jcm9124121)
Supplement: Supplementary file 1 [file jcm-09-04121-s001.pdf]

Supplementary Materials: A Real-World Comparative Analysis of Lenvatinib and Sorafenib as a Salvage Therapy for Transarterial Treatments in Unresectable HCC

Jaejun Lee, Pil Soo Sung, Hyun Yang, Soon Kyu Lee, Hee Chul Nam, Sun Hong Yoo, Hae Lim Lee, Hee Yeon Kim, Sung Won Lee, Jung Hyun Kwon, Jeong Won Jang, Chang Wook Kim, Soon Woo Nam, Si Hyun Bae, Jong Young Choi and Seung Kew Yoon

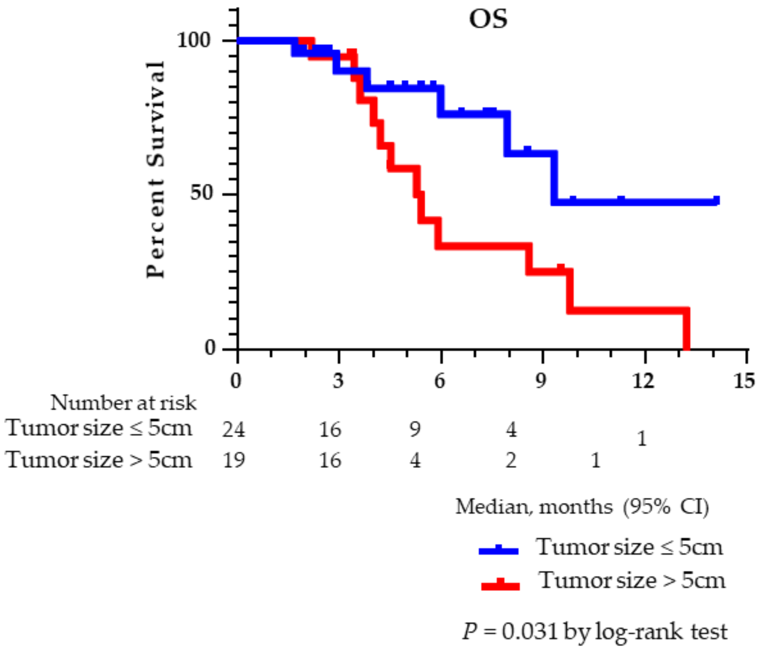

Figure S1. OS of the patients in the lenvatinib group according to the tumor size.

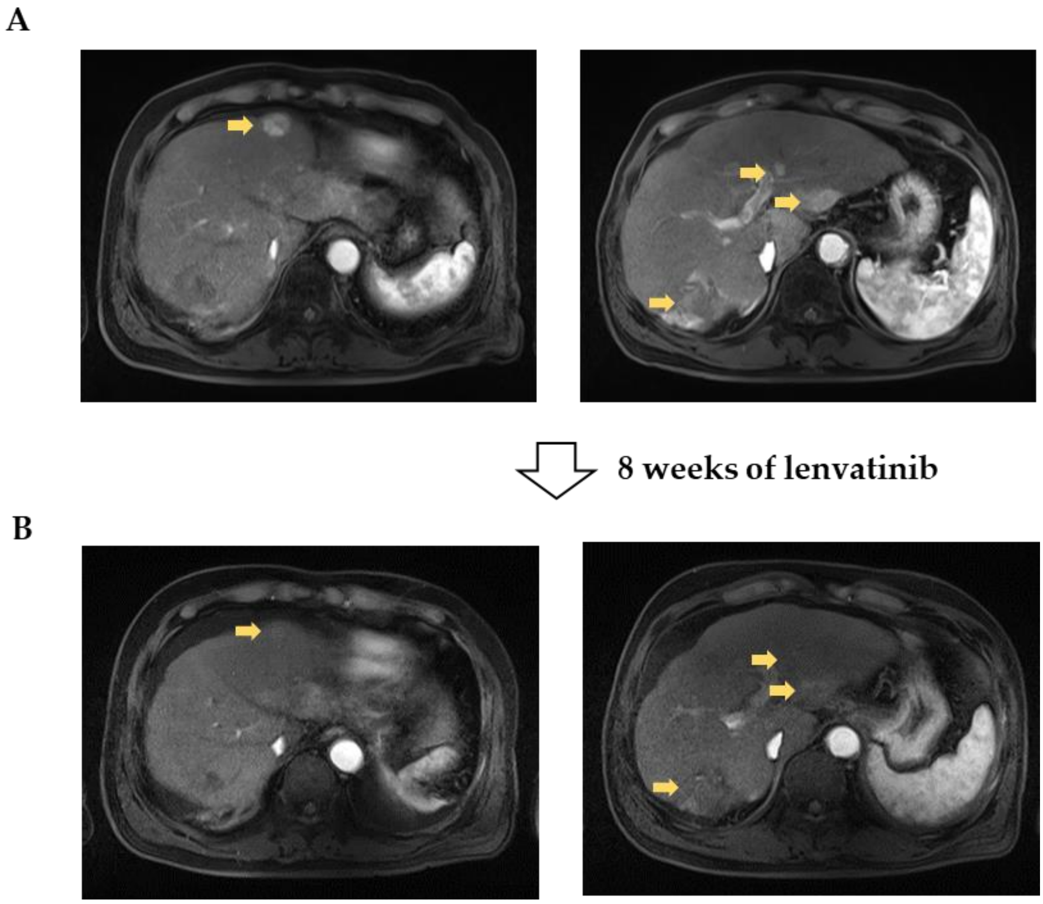

Figure S2. A representative patient case who obtained partial response with lenvatinib after failure of multiple rounds of TACE.
